# A novel *GPR143* duplication mutation in a Chinese family with X-linked congenital nystagmus

**Published:** 2009-04-22

**Authors:** Yuanyuan Peng, Yan Meng, Zheng Wang, Mei Qin, Xiaoqiao Li, Yan Dian, Shangzhi Huang

**Affiliations:** 1 Department of Medical Genetics, Institute of Basic Medical Sciences, Chinese Academy of Medical Sciences, School of Basic Medicine Peking Union Medical College, Beijing, China; 2 WHO Collaborating Centre for Community Control of Hereditary Diseases, Beijing, China; 3 Department of Ophthalmology, Peking Union Medical College Hospital, Beijing, China

## Abstract

**Purpose:**

To elucidate the molecular genetic defect of X-linked congenital nystagmus in a Chinese family.

**Methods:**

Genomic DNA was prepared from peripheral blood. We used allele-sharing analysis to identify the possible locus harboring the disease-causing gene. We screened for mutations in the G protein-coupled receptor 143 gene (*GPR143*) by direct sequencing of the polymerase chain reaction (PCR)-amplified exons.

**Results:**

In analyzing the candidate gene, *GPR143*, in the linked region, a 19 base pair (bp) duplication mutation in exon 1 was detected after direct DNA sequence analysis, which cosegregated in all patients of this family and was present in obligate female carriers.

**Conclusions:**

The identified 19 bp duplication in *GPR143* induces a frame-shift and a premature stop codon, resulting in a truncated protein of 105 residues. These results suggest that this novel mutation is associated with the congenital nystagmus observed in this Chinese family and further support that *GPR143* mutations are the underlying pathogenesis of the molecular mechanism for congenital nystagmus.

## Introduction

Congenital nystagmus (CN) is an ocular hereditary disorder characterized by binocular spontaneous oscillations with onset typically at birth or within the first few months of life [1]. Patients’ oscillations can be horizontal, vertical, torsional, or any combination of these. Reduced vision and poor depth perception are the major symptoms. Congenital nystagmus (frequency of 1/20,000 live births [2]) predominantly occurs secondary to the genetic ocular diseases such as albinism, achromatopsia, and Leber congenital amaurosis. So far, X-linked dominant and X-linked recessive (OMIM 310700), autosomal dominant (OMIM 164100, OMIM 608345, OMIM 193003), and autosomal recessive (OMIM 257400) modes of inheritance have been reported, but X-linked inheritance with incomplete penetrance and variable expressivity is probably the most common. Three different genetic loci for X-linked CN have been mapped to chromosomes Xp11.3–11.4 [3], Xp22 [4], and Xq26-Xq27 [5].

The four-point-one (4.1), ezrin, radixin, moesin (FERM) domain -containing 7 gene (*FRMD7*; OMIM 300628) at Xq26–27 [6] and the G protein-coupled receptor 143 gene (*GPR143*, OMIM 300500) at Xp22 [4] have been identified as disease-causing genes for X-linked CN, but the molecular pathogenic mechanism is largely unknown. To date, multiple mutations of *FRMD7* have been reported. The latter gene, *GPR143*, causes ocular albinism type 1 (OA1), which is an X-linked albinism that mainly effects pigment production in the eye in which affected males show all the ocular signs of albinism including severely impaired visual activity, nystagmus, photophobia, iris transillumination, hypopigmentation of retinal pigmented epithelium, foveal hypoplasia, and misrouting of optic tracts [7]. However, *GPR143* mutations have been identified in two Chinese families with X-linked CN without any classical phenotype of OA1 [4,8].

In this study, we present a four-generation Chinese family with X-linked CN. All affected individuals exhibited nystagmus but without any typical signs of OA1. Upon genetic analysis, we characterized the underlying molecular defect as a novel 19 base pair (bp) duplication in exon 1 of *GPR143*, causing a frame-shift in all affected males. Our results indicate that this novel *GPR143* mutation might be associated with the congenital nystagmus observed in this Chinese family.

## Methods

### Family data

**Figure 1:**
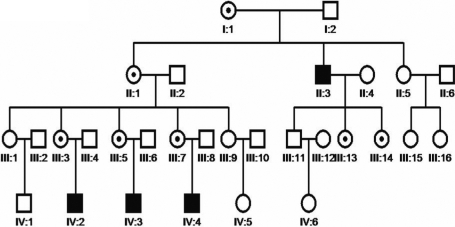
Pedigree of the Chinese family with X-linked congenital nystagmus. Black squares are for affected males, small solid circle within open circles for obligate carrier females, and open symbols for unaffected individuals.

The study had the approval of the Institute of Basic Medical Sciences ethics committee (Beijing, China) and conformed to the tenets of the Declaration of Helsinki. A four-generation Chinese family with X-linked CN was identified in Peking Union Medical College Hospital (Beijing, China) in 1999 [9]. Sample collection was just available from a small part of the whole family (Figure 1). Blood samples were taken after informed written consent from 14 family members including three affected individuals.

### Allele-sharing analysis

Allele-sharing analysis was performed on three affected male individuals with two microsatellite markers (DXS1047 and DXS8071) linked with *FRMD7* and three microsatellite markers (DXS7108, AF003664, and DXS9850) linked with *GPR143*. The primer sequences were taken from the GDB Human Genome database.

### Mutation detection

Genomic DNA was extracted from the peripheral lymphocytes of available subjects. All nine exons of *GPR143* (NM_000273) and part of the flanking intronic regions were amplified by polymerase chain reaction (PCR) from genomic DNA, and each fragment was sequenced directly. PCR primers were designed by the PRIMER3 online software, and the sequences were presented in Table 1.

**Table 1:** Primers and PCR conditions used to amplify genomic segments of *GPR143*.

**Primer name**	**Primer sequence (5′-3′)**	**Annealing temperature (°C)**	**Product size (bp)**
Exon 1-F	AACCTTCCCAACCTTTCTGC	69	698
1-R	CCTCTCGTCCTCACTCCATC		
Exon 2-F	CAGTGAGCAGGGTTTTTACCA	66	537
2-R	AACAGACTCCCAGGGTTTGC		
Exon 3-F	GTCTACCCTGCCGTCTCAAG	66	334
3-R	TGAGCTGCTGTGGATGTTTC		
Exon 4-F	CTCAGCAGCACGAGGAAACT	67	465
4-R	ACAAACGAGAAAGGCAGAGC		
Exon 5-F	CTTAGGGGTCCTCCCATTTC	65	575
5-R	TGGCACTGAGCTAACAAACG		
Exon 6-F	ATCCCCATGGTTGCATAAGA	64	738
6-R	CACATGGTTGGGACATTTCA		
Exon 7-F	GCACCTGGCCCTCTTAGTTT	67	436
7-R	GAGGCCAAGACAGAGGATTG		
Exon 8-F	TTCAGGCACCCTTGAAGGTA	66	539
8-R	CCGGGACAAAGAATCCTCTA		
Exon 9-F	GGCTTGTGTCATCCGTTGTA	61	488
9-R	CCCTTCGGGAAGAAGCTCTA		

The primers were selected inside the flanking introns to amplify the fragments containing coding sequence (exons) and the flanking intronic regions. Primer sequences, size of PCR products, and the annealing temperature for amplification were indicated.

## Results

### Clinical phenotype

In this family, the disease was transmitted from female carrier to affected son and was an apparent X-linked recessive trait (Figure 1), although all sons of the carriers were affected unfortunately. Moreover, female carriers such as III:3, III:5, and III:7 have only one child. If we take a wide view of the intact family pedigree and each of the female carriers could have more offspring, we are likely to expect half of the male offspring of carrier females to be unaffected by the disease. All patients had binocular spontaneous horizontal oscillations with an intermediate frequency (<120 counts per minute) without head nodding. They all had reduced vision, amblyopia, and mild compound hypermetropic astigmatism. However, choroidal vessels could be seen in fundus oculi of two patients with external strabismus (15°−20°) without optic fundi pathological changes or optic nerve lesions.

### Allele-sharing analysis

Allele-sharing analysis of DXS1047 and DXS8071 showed that three patients did not share the same haplotype, which excluded the possibility of mutation in *FRMD7*. However, results of DXS7108, AF003664, and DXS9850 demonstrated that all three affected males obtained the same haplotype and further indicated the linkage of the disease in the family with the mutation in *GPR143*.

### Mutation of *GPR143*

**Figure 2:**
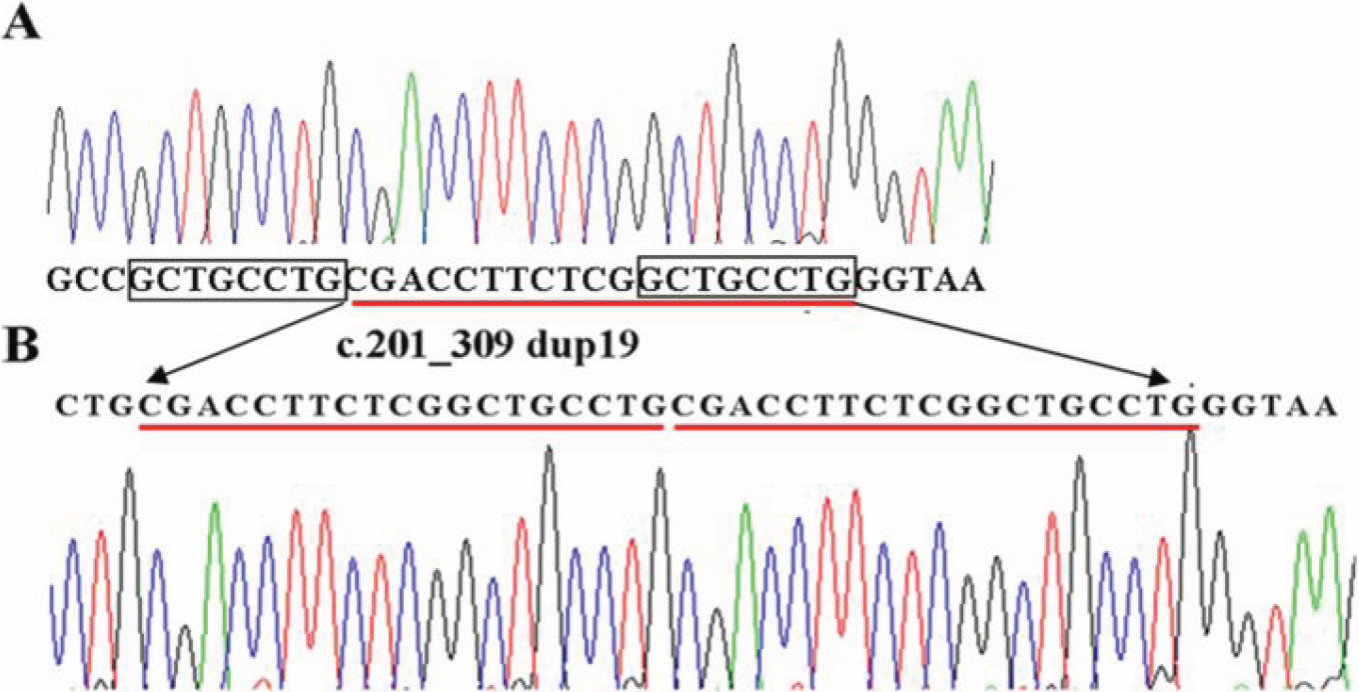
Duplication in *GPR143* identified in subject family with congenital nystagmus. **A**: The sequence for a normal individual shows the wild-type allele. **B**: The sequence for the patient (IV:3) shows the 19-bp (c.291_309) duplication identified in exon 1. We can see that the end part of the duplicated base pairs is the same with the one just ahead of the duplication (shown in the rectangle).

Upon complete analysis of the coding and the adjacent intronic regions of *GPR143,* a previously unidentified 19 bp duplication (c.291_309) mutation in exon 1 was identified in all affected males (Figure 2). This duplication was not detected in normal members of the family or in 100 normal male individuals. It was predicted to result in a frame-shift and a premature stop codon emerged in exon 2, resulting in a truncated protein of only 105 amino acids.

### Carrier identification

**Figure 3:**
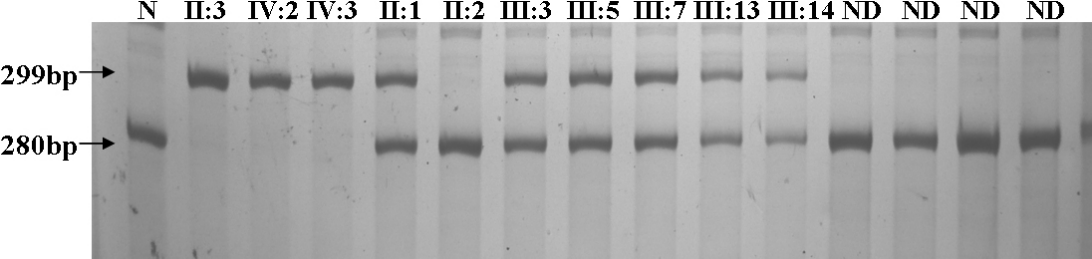
Mutation carriers identified in the subject family with congenital nystagmus. The DNA sequence containing the identified duplication region was amplified by a new pair of primers. Two different allele fragments, a 299 bp PCR fragment that indicated the mutant allele containing the 19 bp duplication and a 280 bp fragment that represented the wild-type allele, would be detected in the female carriers. We can see that all normal individuals obtained only the wild-type alleles of 280 bp, and all male patients only obtained the mutant alleles of 299 bp, which was consistent with the sequence analysis results. All heterozygous individuals (II:1, III:3, III:5, III:7, III:13, and III:14) were determined to be mutation carriers. N: Normal individual; ND: Normal male or female in this family. These individuals (labeled by ND) cannot be identified on the pedigree due to our carelessness and incomplete records of family information during the sample collection, and now we are no longer in touch with them.

A new pair of primers was designed to amplify the mutational region in exon 1. Mutation carriers would obtain two different allele fragments, a 299 bp PCR fragment, which indicated the mutant allele containing the 19 bp duplication, and a 280 bp fragment, which represented the wild-type allele. We could then determine that all heterozygous individuals (II:1, III:3, III:5, III:7, III:13, and III:14) were mutation carriers (Figure 3).

## Discussion

In this study, we report a family with typical clinical signs of X-linked congenital nystagmus. The sequence analysis of *GPR143* identified a novel duplication mutation in exon 1. All affected males were hemizygous for the mutation whereas female carriers were heterozygous for the duplication.

Nystagmus is common in all types of albinism. Diagnosis of the underlying disease often requires extensive clinical, electrophysiological, psychophysical, and eventually molecular genetic examinations, especially when clinical findings are unrevealing. A few individuals initially misdiagnosed with congenital nystagmus have been shown to be affected by ocular albinism type 1 by screening *GPR143* [10,11]. However, in our study, none of the patients with the *GPR143* mutation had the classical phenotype of ocular albinism.

*GPR143* was cloned from the OA1 critical region in Xp22.3–22.2 in 1995 [7]. To date, more than 90 different *GPR143* mutations have since been described. However, there were only nine different *GPR143* mutations identified in the Chinese population, two of which were described in two families with X-linked congenital nystagmus [4,8]. Our case is the third one associated with nystagmus only. Moreover, this c.291_309dup mutation is only the second duplication mutation identified until now. *GPR143* encodes a protein of 404 amino acids containing seven putative transmembrane domains and one potential N-glycosylation site at codon 106 [12]. Defective glycosylation will result in incorrect protein localization [12]. This c.291_309dup mutation leads to a frame-shift and introduces a premature termination codon in exon 2 of *GPR143*. It will result in a predicted protein of 105 amino acids, which just encodes two NH_2_-terminal transmembrane α-helices and loses the potential glycosylation site at codon 106. Therefore, the mutant transcript is likely to be degraded by the nonsense-mediated mRNA decay (NMD) mechanism [13]. It was not verified in our research since we were unable to obtain related RNA of disease tissues.

Two Chinese families with X-linked CN have already been reported to have *GPR143* mutations. One family had a missense mutation [4], which resulted in the substitution of a serine residue with a phenylalanine residue in exon 2. The other had a 37 bp deletion, which caused a frame-shift and resulted in a truncated protein of 93 residues [8]. Moreover, another *GPR143* 14 bp deletion mutation in exon 6 was also identified in white males in a family with X-linked congenital nystagmus [14]. As compared to these findings, the duplication found in our study provides another persuasive evidence of an association between the *GPR143* mutation and CN in the Chinese population. Our results expand the spectrum of clinical phenotypes associated with *GPR143* mutations. Different degrees of phenotypes caused by *GPR143* mutations have been previously reported in other populations [15]. The heterogeneity of the clinical manifestations suggests the existence of modifying genetic, epigenetic, or environmental factors [16]. Perhaps CN is the only phenotype of OA1 in some ethnic populations, and it supports the hypothesis that CN may be a form of ocular albinism [17].

Many *GPR143* gross deletion mutations have been reported, which were believed to be the result of a homologous but unequal crossing-over involving two repetitive sequences such as the Alu family [18-20]. Our finding is only the second duplication mutation so far reported in *GPR143*. We can see the end part of the duplicated base pairs (GCTGCCTG) is the same with the one just ahead the duplication (Figure 2). The first identified duplication mutation, c.163_170dup, was also located in exon 1 of high GC content [18]. The duplicated DNA sequence (GCGGGCCC) is extremely similar to the upstream DNA sequence (GCCGGCCC). Therefore, high similarity between the DNA sequences may be the cause of these mutations, and high GC content is the foundation of the similarity. These mutations might be explained by replication error. However, the possibility of unequal crossing-over cannot be excluded before the real mechanism is disclosed.

Our results provide additional evidence that mutations in *GPR143* are another cause of congenital nystagmus in the Chinese population. Our findings also expand its mutation spectrum and can be useful for gene diagnosis and genetic counseling in Chinese CN patients. We believe that further biochemical studies of the mutations in *GPR143* will yield insight into its molecular mechanism underlying the pathogenesis of congenital nystagmus.
